# Comparison of Presentation and Prognosis of Takayasu Arteritis with or without Stroke or Transient Ischemic Attack—A Retrospective Cohort Study

**DOI:** 10.3390/life12111904

**Published:** 2022-11-16

**Authors:** Durga Prasanna Misra, Upendra Rathore, Prabhaker Mishra, Kritika Singh, Darpan R. Thakare, Manas Ranjan Behera, Neeraj Jain, Manish Ora, Dharmendra Singh Bhadauria, Sanjay Gambhir, Sudeep Kumar, Vikas Agarwal

**Affiliations:** 1 Department of Clinical Immunology and Rheumatology, Sanjay Gandhi Postgraduate Institute of Medical Sciences (SGPGIMS), Lucknow 226014, India; 2 Department of Biostatistics and Health Informatics, Sanjay Gandhi Postgraduate Institute of Medical Sciences (SGPGIMS), Lucknow 226014, India; 3 Department of Nephrology, Sanjay Gandhi Postgraduate Institute of Medical Sciences (SGPGIMS), Lucknow 226014, India; 4 Department of Radiodiagnosis, Sanjay Gandhi Postgraduate Institute of Medical Sciences (SGPGIMS), Lucknow 226014, India; 5 Department of Nuclear Medicine, Sanjay Gandhi Postgraduate Institute of Medical Sciences (SGPGIMS), Lucknow 226014, India; 6 Department of Cardiology, Sanjay Gandhi Postgraduate Institute of Medical Sciences (SGPGIMS), Lucknow 226014, India

**Keywords:** mortality, prognosis, stroke, survival, Takayasu arteritis, transient ischemic attack

## Abstract

Takayasu arteritis (TAK) could cause a stroke or transient ischemic attack (TIA) in young individuals due to inflammatory vascular occlusion or intracerebral hemorrhage. We compared the clinical presentation, angiographic features, longitudinal patterns of disease activity, medical treatments, and survival in 34 TAK patients with stroke/TIA and 157 without stroke/TIA from a single-center retrospective cohort. TAK patients with stroke/TIA were older (*p* = 0.044) with a greater proportion of males (*p* = 0.022), more frequent vision loss (odds ratio (OR) for stroke/TIA vs. without stroke TIA 5.21, 95% CI 1.42–19.14), and less frequent pulse or blood pressure inequality (OR 0.43, 95% CI 0.19–0.96) than TAK patients without stroke/TIA. Hata’s angiographic type IIa was more common in TAK patients with stroke/TIA (OR 11.00, 95%CI 2.60–46.58) and type V in TAK patients without stroke/TIA (OR 0.27, 95% CI 0.12–0.58). Cyclophosphamide was used more often in TAK patients with stroke/TIA (*p* = 0.018). Disease activity at baseline, 6, 12, and 24 months of follow-up was mostly similar for both groups. Risk of mortality was similar in TAK patients with or without stroke/TIA (hazard ratio unadjusted 0.76, 95% CI 0.15–3.99; adjusted for gender, age of disease onset, delay to diagnosis, baseline disease activity, and the number of conventional or biologic/targeted synthetic immunosuppressants used 1.38, 95% CI 0.19–10.20) even after propensity score-matched analyses. Stroke or TIA does not appear to affect survival in TAK patients adversely.

## 1. Introduction

Takayasu arteritis (TAK) is a large vessel vasculitis (LVV) more common in younger females and Asian countries [1,2,3]. Unlike the counterpart LVV of Giant Cell Arteritis (GCA), which commonly affects the extracranial branches of the carotid artery, TAK frequently affects intracranial branches of the carotid artery [4]. 

Inflammatory vascular occlusion in TAK patients is usually insidious in onset. Gradually progressive vascular fibrosis allows for the development of extensive collateral circulation to compensate for the reduced vascular flow. Such individuals might be diagnosed with TAK when incidentally detected to have absent pulses, asymmetric blood pressure between the upper or lower limbs, or vascular bruits. Inadequate restoration of distal blood supply with collaterals results in claudication pain in the upper or lower limbs [5,6]. However, vascular occlusion of abrupt onset can result in severe downstream ischemia resulting in cardiovascular events, viz., ischemic stroke or myocardial infarction [1,3,7], even in the absence of preceding constitutional symptoms [7]. The risk of stroke is increased with TAK. A study from a primary care database from the United Kingdom compared 142 TAK patients (13.4% with stroke) with 1371 age- and sex-matched controls (4.9% with stroke). The risk of stroke was significantly higher in TAK patients than in control subjects (hazard ratio 4.38, 95% confidence interval (95% CI) 2.24–8.55) [8]. Another study of a nationwide health insurance database from South Korea reported that the standardized incidence ratio of stroke was higher for TAK patients when compared with the population (7.39, 95% CI 5.79–9.29), similar for both men and women [9]. 

Up to one-third of TAK patients present with major ischemic events (including stroke) [7,10,11], even with normal acute-phase reactants [11]. Patients with TAK can have recurrent strokes [12]. Ischemic stroke in TAK patients can also occur following vascular procedures [13]. Hypertension is noted in up to 75–80% of TAK cases [14,15]. Uncontrolled hypertension can result in hemorrhagic stroke [3,14,15]. From a series of six individuals with TAK (mean age 32.2 years) with neurological symptoms, four had stroke and one had a transient ischemic attack (TIA) [16]. Thus, TAK is an important cause of stroke in the young [17]. Stroke or TIA occurs in similar proportions of TAK with onset in childhood or in adulthood [18,19]. Stroke in TAK patients can have fatal consequences [20,21]. Analysis of a national health insurance database from South Korea revealed that 38 (12.8%) of the 298 deaths reported in 2731 patients with TAK were due to stroke [22]. Janus kinase inhibitors are increasingly used in the management of inflammatory rheumatic diseases including TAK [23]. Recent studies have raised concerns about the risk of stroke and other cardiovascular events with Janus kinase inhibitors [24].

Stroke and TIA can occur due to the involvement of either extracranial or intracranial vessels in TAK cases. From a series of 20 patients with TAK from North America, 6 had a stroke, and 4 had involvement of intracranial vessels in vascular imaging [25]. From another series of 190 patients with TAK from South Korea, 21 (11%) had ischemic stroke. Among these, intracranial arterial stenosis was evident in eleven, whereas extracranial vessels (carotid or subclavian arteries) were involved in fifteen patients [26]. 

Stroke in TAK patients can be an indication as well as a consequence of vascular procedures (both open surgical and endovascular). Among 40 patients with TAK undergoing 98 surgical procedures, 8 of the 64 initial procedures and another 8 of the 34 repeat procedures were for cerebrovascular insufficiency [27]. From another series of 42 patients with TAK who underwent 60 vascular surgeries, strokes (albeit minor) were observed in 2 patients [13]. Stroke is an important complication after vascular surgery. Out of 11 observed complications following 154 procedures undertaken in 116 patients with TAK, stroke was responsible for 3 out of 6 complications during open surgical repair and 2 out of 5 complications during endovascular repair [28]. Stroke can occur both as an early or delayed complication after vascular surgery. In a cohort of 79 TAK patients undergoing 166 procedures (104 open surgical and 62 endovascular), 4 strokes were among the 70 complications observed. One stroke resulted in death. One stroke was observed as an early complication (occurring within 30 days of the procedure) following endovascular procedures, whereas three strokes occurred as delayed complications (occurring beyond 30 days after the procedure) following open surgical procedures [29]. A systematic review and meta-analysis of 12 studies comparing 261 patients with TAK undergoing endovascular procedures with another 338 undergoing open surgical procedures reported lower odds of stroke with endovascular than with open surgical procedures (odds ratio 0.33, 95% CI 0.12–0.90) [30]. 

Stroke in the context of vasculitis is associated with adverse implications. A study of 26,855 adults with systemic vasculitis from the National Inpatient database from the United States of America (USA) reported stroke or cerebrovascular disease in 2.57% of these patients. Significant loss of functional capacity was more likely in patients with vasculitis with stroke (79.9%) than those without stroke (69.7%). Those with vasculitis with stroke had a longer hospital stay (12.9 days) than those without (8.1 days) along with higher costs of hospitalization (USD 169,440 vs. USD 111,537). Vasculitis with stroke had a more frequent transfer to a hospital care facility after discharge (8.2%) than those without stroke (4.6%). Deaths were similar in those with vasculitis with or without stroke (3.7% vs. 3.5%) [31].

Overall, the literature on stroke/TIA in TAK patients is scant. Few studies have compared TAK with or without stroke/TIA [9,32,33,34]. Even fewer studies have compared TAK with or without stroke/TIA in a matched analysis [32]. The objectives of the present study were to report the characteristics of patients with TAK who developed stroke (ischemic or hemorrhagic) or TIA at presentation or on follow-up in a large retrospective cohort of patients with TAK from India. Furthermore, we have compared the clinical presentation, angiographic features, disease course on follow-up, and therapies received by patients with or without stroke/TIA. We have also compared the survival of patients with TAK with or without stroke/TIA using unadjusted as well as adjusted and propensity score matched analyses accounting for various important prognostic factors. 

## 2. Materials and Methods

### 2.1. Selection of Patients for the Study

This was a retrospective cohort study involving a chart review of patients with TAK attending a dedicated vasculitis clinic at the Department of Clinical Immunology and Rheumatology at Sanjay Gandhi Postgraduate Institute of Medical Sciences (SGPGIMS), Lucknow, a large tertiary-care referral and training center in North India. The study included adult patients with TAK fulfilling either the American College of Rheumatology 1990 classification criteria [35] or the Chapel Hill Consensus Conference 2012 definition for TAK [36]. Pediatric patients with TAK fulfilled the 2008 European Alliance of Associations for Rheumatology/Pediatric Rheumatology European Society/Pediatric Rheumatology International Trials Organization classification criteria for TAK [37]. Patients with stroke (ischemic or hemorrhagic or a clinical syndrome compatible with stroke but without available imaging) or TIA at initial presentation or on follow-up were identified. 

### 2.2. Clinical Characteristics

The demographic features (gender, age at cohort entry, delay to diagnosis) and any comorbid conditions recorded in the clinic files were noted. Clinical features at presentation (constitutional, cardiovascular, neurological, abdominal, and miscellaneous), inflammatory markers at presentation (erythrocyte sedimentation rate (ESR), C-reactive protein (CRP), neutrophil: lymphocyte ratio), and diagnostic imaging modalities used during the initial evaluation (computerized tomographic angiography (CTA), magnetic resonance angiography (MRA), 18-fluorodeoxyglucose positron emission tomography computerized tomography (PET-CT), color Doppler ultrasound, conventional angiography) were recorded. A single patient could have undergone evaluation with multiple imaging modalities. The individual vessels involved in angiography as well as the overall angiographic subtype of TAK as per Hata’s classification were recorded. As per this angiographic classification of TAK, type I disease refers to involvement of the branches of the arch of aorta; type IIa refers to the involvement of the ascending aorta, arch of aorta, and its branches; type IIb refers to the involvement of the segments in IIa along with the descending thoracic aorta; type III refers to the involvement of descending thoracic aorta and abdominal aorta (may involve the renal arteries); type IV refers to the involvement of the abdominal aorta or renal arteries alone; and type V disease has pan-aortic involvement with features of types IIb and IV disease. Pulmonary artery (P+) or coronary artery involvement (C+), if present, are denoted in addition [38].

### 2.3. Treatments Received and Longitudinal Patterns of Disease Activity

Immunosuppressive therapies (glucocorticoids or disease-modifying antirheumatic drugs (DMARDs)) received by the patients with TAK as first-line or subsequently on follow-up were recorded. Disease activity was recorded using the Disease Extent Index in TAK (DEI.TAK) [39], the Indian TAK Clinical Activity Score (ITAS2010) [40], and the physician global assessment (PGA) of active or inactive disease at baseline and follow-up visits, and using the National Institutes of Health (NIH) disease activity criteria for follow-up visits [20]. For this analysis, disease activity was recorded at the initial presentation and subsequently at 6 months, 12 months, and 24 months. Dates of the first visit and the last follow-up, as well as survival at the last follow-up, were recorded. 

### 2.4. Statistical Analysis including Survival Analysis

Comparisons were performed between patients with TAK with stroke or TIA versus TAK without stroke or TIA. Continuous variables were summarized using means (± standard deviations (SD)) and compared between groups using an unpaired Student’s t-test. Categorical variables were summarized using numbers and percentages and compared using the Chi-square test (if each of the four cells had at least five observations) or Fisher’s exact test. Odds ratios (with 95% CI) comparing TAK patients with stroke or TIA with those without stroke/TIA were calculated using univariable binary logistic regression. Since TAK is a rare disease, a sample size was not calculated for the study. 

Survival in the cohort was analyzed by censoring the data at the last follow-up visit or at the time of death. For those TAK patients who had stroke/TIA at or before the first visit, the first visit was taken as the time of cohort entry. For those TAK patients who did not have stroke/TIA at the first visit but developed subsequently on follow-up, the date of stroke was taken as the time of cohort entry. The hazard ratio for mortality between patients with stroke or TIA compared with those without stroke or TIA was calculated. For adjustment, six prognostic variables were chosen a priori, viz., gender, age at disease onset, delay to diagnosis, disease activity status at baseline (active or inactive), the number of conventional DMARDs used, and the number of biologic DMARDs used [19,41,42,43,44,45]. A crude hazard ratio as well as hazard ratios adjusted for these prognostic variables were calculated using Cox regression analysis. Patients with stroke or TIA were matched with those without stroke/TIA using propensity scores generated on these prognostic variables, with a tolerance of 0.1 for propensity score matching (using the Statistical Package for Social Sciences version 23). Kaplan–Meier plots were synthesized separately for the overall cohort and the propensity-score-matched cohort. Survival between patients with stroke/TIA and without were compared using the log-rank test. All analyses were performed using STATA 16.1 I/C unless otherwise indicated. Statistical significance was set at a 5% level of difference. 

### 2.5. Ethics Committee Approval 

The Institute Ethics Committee of SGPGIMS, Lucknow approved the study (Institute Ethics Committee Code 2021-165-IP-EXP-40, date of approval 16 July 2021). Because of the retrospective review of medical records without any direct contact with patients, the ethics committee provided a waiver of written informed consent, in line with the prevalent local regulations. 

## 3. Results

### 3.1. Characteristics of Patients with TAK with Stroke/TIA

Of the 191 patients with TAK in the cohort, 34 (17.8%) had developed stroke or TIA (thirty-two strokes, two TIA). Of the 32 patients with stroke, 27 had an ischemic stroke, 4 had a hemorrhagic stroke, and the type of stroke was unclear in 1 patient (underlying aortic regurgitation with difficult-to-treat hypertension, developed hemiparesis, and died before imaging could have been performed). Twenty-nine patients (15.2%) had a stroke at or before the initial presentation of TAK, whereas three others developed stroke on follow-up. A total of 3 of the 32 TAK patients with stroke (9.4%) had recurrent ischemic strokes. None of the strokes were attributed by the treating physician to causes other than TAK such as surgical or endovascular procedures, or to atherosclerosis.

### 3.2. Characteristics of Patients with Stroke/TIA Compared with Those without Stroke/TIA

Patients with TAK with stroke/TIA were older (mean age 28.65 years vs. 24.74 years without stroke). Although both groups had a predominance of females, TAK without stroke/TIA comprised a significantly greater proportion of females. The delay to diagnosis and the duration of follow-up was similar for TAK with or without stroke/TIA. Diabetes mellitus was more prevalent in TAK with stroke/TIA than in those without, whereas other comorbidities were similar (Table 1). 

### 3.3. Clinical and Laboratory Features at Presentation of TAK Patients with Stroke/TIA Compared with Those without Stroke/TIA

Loss of vision was more frequent in TAK patients with stroke/TIA when compared to those without (odds ratio 5.21. 95%CI 1.42–19.14). Inequality or asymmetry of pulse or blood pressure between the upper or lower limbs was less frequently observed in TAK patients with stroke/TIA than those without (odds ratio 0.43, 95%CI 0.19–0.96). All the other clinical features were not significantly different between the two groups. TAK patients with stroke or TIA had a lower ESR at baseline than those without stroke/TIA. CRP and neutrophil: lymphocyte ratio were similar between the groups at baseline (Table 2). Hemoglobin was higher in patients with TAK/stroke than those without.

Overall, few patients (7/34, 20.5%) with stroke had been tested for antiphospholipid antibodies. IgM anticardiolipin antibodies (none out of six), IgG anticardiolipin antibodies (none out of seven), anti-beta2 glycoprotein antibodies (none out of six), and lupus anticoagulant (none out of two) were undetectable in all of those tested.

### 3.4. Vascular Imaging at Presentation of TAK Patients with Stroke/TIA Compared with Those without Stroke/TIA

Patients with stroke had more frequently undergone PET-CT during the initial assessment (odds ratio 2.14, 95%CI 1.01–4.57). Similar proportions of patients had undergone CTA, MRA, conventional angiography, or color Doppler ultrasound (Table 3).

Hata’s angiographic subtype IIa was more frequently observed in TAK patients with stroke/TIA, whereas Hata’s subtype V was more common in TAK patients without stroke/TIA (Table 3). Concerning the involvement of individual arteries, the right subclavian artery and the right carotid artery were more frequently involved in TAK patients with stroke/TIA, whereas the descending thoracic aorta, abdominal aorta, or celiac trunk were more commonly involved in TAK patients without stroke/TIA (Supplementary Table S1).

### 3.5. Medical Treatments Received among TAK Patients with Stroke/TIA Compared with Those without Stroke/TIA

Similar proportions of patients with TAK with stroke/TIA or without had been treated with glucocorticoids or with the conventional DMARDs methotrexate, leflunomide, azathioprine, mycophenolate, or tacrolimus. Cyclophosphamide, while overall used in four patients, had been used more frequently in patients with stroke. Few patients in our cohort were on biologic DMARDs (adalimumab, tocilizumab) or targeted synthetic DMARDs (tofacitinib), the proportions of which were not significantly different between TAK patients with or without stroke/TIA. A significantly higher proportion of patients with TAK with stroke/TIA were on statins, aspirin, or clopidogrel (Supplementary Table S2).

Methotrexate and tacrolimus were the most commonly used first-choice DMARDs in both TAK patients with or without stroke/TIA. Regarding the prevalent choices of second-line DMARDs, methotrexate and azathioprine had been more frequently used in TAK patients with stroke/TIA, whereas mycophenolate and azathioprine had been used more often in TAK patients without stroke/TIA (Table 4).

### 3.6. Surgical or Endovascular Procedures among Those TAK Patients with Stroke/TIA Compared with Those without Stroke/TIA

The proportions of TAK patients undergoing surgical or endovascular procedures related to TAK were similar between those with or without stroke/TIA (odds ratio 0.91, 95%CI 0.35–2.29). Six patients (17.65%) with TAK with stroke/TIA had undergone a total of nine procedures. Amongst those TAK patients without stroke or TIA, thirty patients (19.10%) had undergone thirty-nine procedures. None of our patients had developed a stroke after procedures related to TAK.

### 3.7. Longitudinal Patterns of Disease Activity among TAK Patients with Stroke/TIA Compared with Those without Stroke/TIA

At the initial presentation, similar proportions of TAK patients with or without stroke/TIA had active disease as per PGA. The baseline disease activity scores, DEI.TAK and ITAS2010, were also similar between the two groups. At 6 months, 12 months, and 24 months of follow-up, the DEI.TAK, the ITAS2010, and the proportions of patients with active disease as per PGA remained similar between TAK patients with stroke/TIA or without. At 6 months and 24 months, the NIH disease activity scores were similar between TAK patients with stroke/TIA or without. However, at 12 months, the NIH disease activity scores were lower for TAK patients with stroke/TIA than those without (Table 5).

### 3.8. Comparison of Mortality between Patients with TAK with or without Stroke/TIA

#### 3.8.1. Unadjusted Survival Analyses for TAK Patients with Stroke/TIA

**Figure 1:**
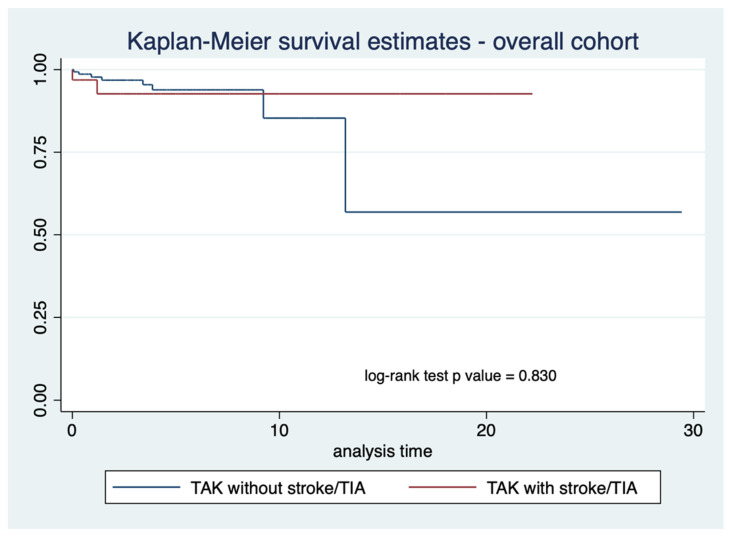
Kaplan–Meier curve comparing survival in Takayasu arteritis (TAK) patients with or without stroke or transient ischemic attack (TIA) for the entire cohort (*n* = 184 with available follow-up visits).

**Figure 2:**
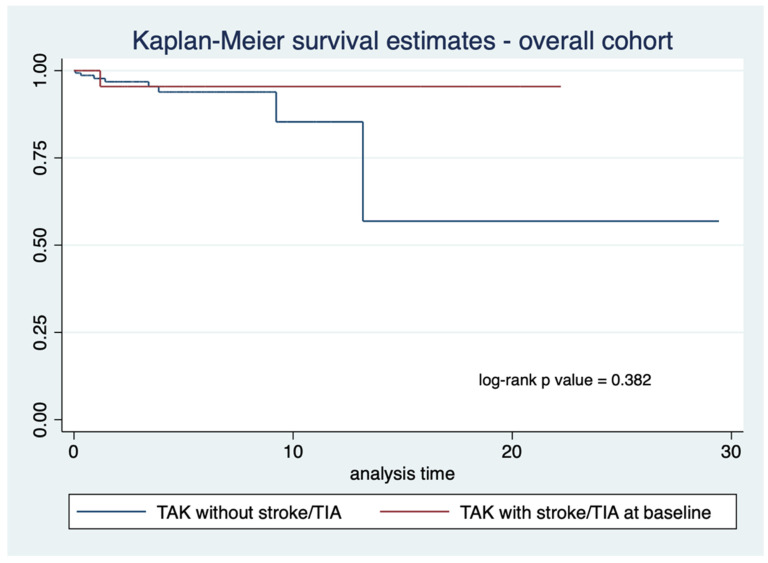
Kaplan–Meier curve comparing survival in Takayasu arteritis (TAK) patients with or without stroke or transient ischemic attack (TIA) for those TAK patients with stroke or TIA at presentation (*n* = 181 with available follow-up visits).

The proportions of recorded deaths were similar between TAK patients with stroke/TIA (2 out of 34) or without stroke/ TIA (8 out of 157) (Table 1). Kaplan–Meier survival curves for the 182 patients with follow-up data revealed similar survival for TAK patients with or without stroke/TIA at any time (log-rank test *p*-value 0.830) (Figure 1). Similar results were observed even after limiting this analysis to TAK patients with stroke or TIA at or before the initial presentation (log-rank test *p*-value 0.382) (Figure 2).

#### 3.8.2. Hazard Ratios for TAK Patients with Stroke/TIA Compared with Those without

The unadjusted hazard ratio for mortality for TAK patients with stroke/TIA was 0.76 (95%CI 0.15–3.99). Even after adjusting for gender, age at disease onset, delay to diagnosis, disease activity at baseline, the number of conventional DMARDs, and the number of biological DMARDs used, the mortality rate for TAK patients with stroke/TIA continued to be similar when compared with TAK patients without stroke/TIA (hazard ratio 1.38, 95%CI 0.19–10.20) (Table 6). Similar results were observed even after limiting this analysis to TAK patients with stroke or TIA at or before the initial presentation (unadjusted hazard ratio for mortality with stroke/TIA 0.39, 95%CI 0.05–3.40, hazard ratio for mortality adjusted for prognostic variables 0.42, 95%CI 0.03–5.90).

#### 3.8.3. Propensity Score-Matched Cohort Analysis Comparing Survival among TAK Patients with or without Stroke/TIA

**Figure 3:**
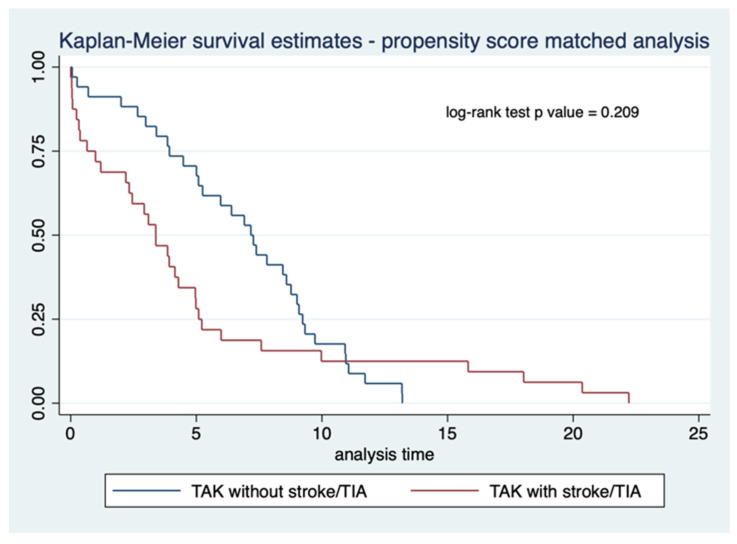
Kaplan–Meier curve comparing survival in Takayasu arteritis (TAK) patients with or without stroke or transient ischemic attack (TIA) for 34 pairs matched using propensity scores for gender, age of disease onset, delay to diagnosis, disease activity at baseline, number of conventional, biologic, or targeted synthetic disease-modifying anti-rheumatic drugs used (34 matched pairs with or without stroke/TIA).

Propensity score matching was performed based on gender, age at disease onset, delay to diagnosis, disease activity at baseline, the number of conventional DMARDs, and the number of biological DMARDs used. With a tolerance of 0.1, 34 matched pairs of patients with TAK with or without stroke/TIA were identified. Similar survival was observed between the matched pairs of TAK patients with or without stroke/TIA using Kaplan–Meier survival curves (log-rank test *p*-value 0.209) (Figure 3).

### 3.9. Comparison of Findings of the Present Study with Other Studies

The findings of the other available studies comparing patients with TAK with or without stroke [9,32,33,34] as well as of this study are summarized in Supplementary Table S3.

## 4. Discussion

### 4.1. Summary of the Findings of the Study

In this large retrospective cohort of patients with TAK from India, those with a stroke or TIA at baseline or subsequently on follow-up were older with a relatively greater proportion of males than those without stroke/TIA. TAK patients with stroke/TIA more often had vision loss and less frequently had an inequality of pulse or blood pressure in the limbs. Hata’s angiographic type IIa was more commonly observed in TAK patients with stroke/TIA, as opposed to type V in TAK patients without stroke or TIA. Apart from the more frequent use of cyclophosphamide in TAK patients with stroke/TIA, immunosuppressive therapies had been used similarly in both groups. Survival analyses, crude as well as adjusted for important prognostic factors, revealed similar survival in patients with TAK with or without stroke/TIA. The lack of difference in survival between TAK patients with or without stroke/TIA was also confirmed after propensity score matching for the same prognostic variables. We could only identify four other studies which had compared TAK patients with or without stroke/TIA [9,32,33,34].

### 4.2. Stroke at Presentation and on Follow-Up in TAK Patients

Fifteen percent of TAK patients in our cohort had stroke at or before presentation. The overall prevalence of stroke or TIA in our cohort was 17.8%. Hoffmann et al. reported stroke in 20.4% of a cohort of TAK patients from South Africa [46]. Mirouse et al. reported stroke or TIA in 19.7% of TAK patients from a multicentric cohort study from France (32% had stroke preceding the diagnosis of TAK) [34]. de Paula from Brazil reported stroke in 15% of TAK patients (14/18 at presentation, 4/18 on follow-up) [47]. Arnaud et al. from France reported ischemic stroke or TIA in 15.9% of their TAK patients [48]. Some other cohorts of TAK patients have reported stroke less frequently. Li-xin et al. from China reported ischemic stroke in 9.5% [49], Soto et al. from Mexico in 9% [50], Yang et al. from China in 4.9% [51], and Couture et al. in 13.5% of their cohort of TAK patients [32]. Hwang et al. reported ischemic stroke in 11% of their cohort of TAK patients from South Korea [26]. Fan et al. from China reported stroke in 6% of their cohort of pediatric TAK patients [52]. On the other hand, Kong et al. from China reported stroke in 34.4% of their patients with TAK [33]. A systematic review of 21 studies reported stroke or TIA in 15.8% (95%CI 10.7–22.6) of TAK patients with a high degree of heterogeneity in the pooled estimates (I^2^ 94%). Of the 18 studies reporting stroke alone, the pooled prevalence of stroke was 11.7% (95%CI 10.1–13.5) without considerable statistical heterogeneity (I^2^ 8.3%) [53]. Another systematic review reported stroke or TIA in 11.7% of patients (95%CI 9.2–14.1) (33 studies, I^2^ 77.3%), and stroke alone in 8.9% (95%CI 7.0–10.9) (29 studies, I^2^ 64.9%), with considerable statistical heterogeneity in both these estimates [54]. Stroke at or preceding diagnosis was reported in 13.1% of patients (95%CI 7.6–18.6) (three studies, I^2^ 33%) [54]. We recorded only three strokes during follow-up in those without stroke at presentation. The prevalent literature suggests that a greater proportion of TAK patients than we observed have stroke on follow-up (pooled prevalence 9.2%, 95%CI 3.7–14.7 %, seven studies, I^2^ 57.4%) [54]. The systematic review by Duarte et al. supports our observation that ischemic stroke is more common than hemorrhagic stroke in TAK patients [53].

### 4.3. Recurrent Strokes in TAK Patients

Recurrent strokes were observed in 9.4% of our TAK patients with stroke, similar to Yang et al. (10%) [51]. However, other series have reported recurrent strokes more often. Kong et al. reported recurrent strokes in 21.4% of patients [33], and Mirouse et al. reported recurrent strokes in 54.1% of TAK patients [34]. Mirouse et al. also reported a significantly greater risk of recurrent stroke in those TAK patients with a previous stroke (hazard ratio 5.11, 95%CI 2.91–8.99) [34]. All these studies had a similar follow-up duration to our study, therefore, factors other than follow-up duration are likely responsible for such discrepancies observed in recurrent strokes.

### 4.4. Demographic Features of TAK Patients with Stroke/TIA Compared with Those without

Couture et al. [32], Ahn et al. (from South Korea) [9], and Mirouse et al. [34] reported that TAK patients with stroke were older than those without stroke, similar to our findings. In the population-based national insurance database study from South Korea, age was the only factor associated with increased risk of stroke in TAK patients (hazard ratio 1.02, 95%CI 1.00–1.04 per year) after multivariable adjustment [9]. On the other hand, Kong et al. reported a younger age of TAK patients with cerebral infarction than those without [33]. While overall TAK was more common in females than in males both in those with or without stroke/TIA, we observed a significantly greater proportion of males with TAK with stroke/TIA. Similar observations were also reported by Kong et al. [33] and Mirouse et al. [34], whereas Ahn et al. reported a slightly lesser proportion of males than females among TAK patients with stroke [9].

### 4.5. Disease Activity among TAK Patients with Stroke/TIA Compared with Those without

We observed that clinically assessed disease activity of TAK (using ITAS2010, DEI.TAK, or NIH criteria), CRP, and neutrophil: lymphocyte ratio at presentation were similar at presentation in those TAK patients with stroke/TIA when compared with those without stroke/TIA. ESR was lower in TAK patients with stroke/TIA than in those without. Ahn et al. reported numerically higher (but not statistically different) levels of ESR and CRP in TAK patients with stroke than those without [9]. Kong et al. also reported similar ESR and CRP levels in TAK patients with or without cerebral infarction. However, they also reported higher ITAS2010 scores in TAK patients with cerebral infarction [33]. Such inconsistent results are likely because the assessment of disease activity in TAK patients is challenging. ESR and CRP inconsistently associate with active TAK [55,56]. The ITAS2010 score has a poorer association with PGA at the baseline visit than on subsequent visits [56]. Given the limitations of the currently available disease activity measures for TAK [56], we assessed disease activity using multiple measures and at multiple time points. Other than TAK patients with stroke/TIA having lower NIH disease activity scores at 12 months (but not at other time points), no differences were evident concerning longitudinal disease activity in TAK patients with or without stroke/TIA.

### 4.6. Comorbidities in TAK Patients with Stroke/TIA Compared with Those without

We observed that TAK patients with stroke or TIA in our cohort more frequently had diabetes than those without, whereas other traditional cardiovascular risk factors were similar. Couture et al. [32], Ahn et al. [9], and Hwang et al. [26] noted a similar prevalence of traditional cardiovascular risk factors in patients with TAK with or without stroke. Fan et al. reported similar proportions of stroke in children with TAK with or without hypertension [52]. Kong et al. reported a greater prevalence of dyslipidemia in TAK patients with cerebral ischemia than those without [33]. Mirouse et al. reported that smoking was more often observed in TAK patients with stroke or TIA than those without [34]. Interestingly, they observed that TAK patients with a prior myocardial infarction had a lesser risk of stroke or TIA [34].

### 4.7. Clinical Presentation and Risk Factors for TAK Patients with Stroke/TIA

Loss of vision was observed more frequently, whereas asymmetry of pulse or blood pressure was less often noted in our TAK patients with stroke or TIA than without. The delay to diagnosis was similar in both groups. Other studies have reported contrary findings. Mirouse et al. reported a greater risk of stroke in TAK patients with a delay to diagnosis exceeding one year (hazard ratio 2.22, 95%CI 1.30–3.80) [34]. Kong et al. reported blurring of vision (but not loss of vision) and syncope more frequently in TAK patients with cerebral ischemia than those without. Further, they identified hyperlipidemia, higher baseline values of ITAS2010, a greater number of involved arteries, a greater number of occluded arteries, and middle cerebral artery involvement as predictive factors for stroke in TAK patients [33]. The risk of stroke in TAK patients might also vary with ethnicity. Arnaud et al. reported that stroke was more common in TAK patients of African ancestry (25%) than those of Caucasian ancestry (5.1%) [48]. Antiphospholipid antibodies predispose to thrombotic events including stroke, particularly in the context of inflammatory rheumatic diseases [57]. We could not detect antiphospholipid antibodies in our TAK patients with stroke, albeit few had been tested for the same. Couture et al. [32] and Kong et al. [33] reported antiphospholipid antibodies in 18% and 10% of their patients with TAK with stroke, respectively. Contrary to this, antiphospholipid antibodies were reported in ten out of twenty-two TAK patients by Jordan et al. from a cohort in the United Kingdom [58]. From this cohort, vascular complications were noted in 7/10 TAK patients (stroke in 4/10) with antiphospholipid antibodies as opposed to 3/12 without [58]. The greater prevalence of antiphospholipid antibodies in this cohort might relate to the reporting center being a tertiary care referral center for antiphospholipid antibody syndrome [58].

### 4.8. Angiographic Involvement in TAK Patients with Stroke/TIA

We observed a significantly greater proportion of Hata’s type IIa with more frequent involvement of the right carotid and right subclavian arteries in TAK patients with stroke/TIA. Kong et al. reported similar proportions of type V and type I disease in TAK patients with or without cerebral ischemia. While the involvement of extracranial arteries was similar, intracranial arteries were more commonly involved in TAK patients with cerebral ischemia than without [33]. Mirouse et al. noted similar distributions of Hata’s angiographic subtypes in TAK patients with or without stroke or TIA. Similar to our observation, the supra-aortic trunk was more frequently involved, and contrary to our observation, the thoracic aorta was less frequently involved in TAK patients with stroke or TIA [33]. de Paula et al. reported the involvement of branches arising from the aortic arch in 55% of their TAK patients with stroke [47]. Couture et al. observed a greater frequency of internal carotid artery involvement in TAK patients with stroke than those without stroke [32].

### 4.9. Treatments Used in Patients with TAK with Stroke/TIA

The use of immunosuppressive treatments in patients with TAK with stroke has been scarcely reported. Since TAK is corticosteroid-responsive, patients with TAK with stroke with active disease are generally treated with corticosteroids and followed up with DMARDs for maintenance of immunosuppression [23,59,60]. Ringleb et al. from Germany [61] and Couture et al. [32] reported treatment with corticosteroids in all their TAK patients with stroke. Couture et al. further reported the use of high-dose pulsed intravenous corticosteroids in 6/17, methotrexate in 7/17, and tocilizumab in 2/17 TAK patients with stroke [32]. Kong et al. reported the use of corticosteroids in 86% and immunosuppressive therapies in 81% (biologic DMARDs in 5%) of their TAK patients with stroke [33]. Other studies have reported the use of immunosuppressive therapies in fewer patients. Mirouse et al. reported the use of corticosteroids in 52% and immunosuppressive therapies in 41% (biologic DMARDs in 6%) of their TAK patients with stroke [34]. In our cohort, corticosteroids and DMARDs had been used in ~80% of TAK patients with stroke. Porter et al. from the United Kingdom reported medical therapies in 8 TAK patients with cerebral ischemia among their 145 patients with TAK. Seven out of eight of these patients were initially treated with pulsed intravenous methylprednisolone followed by oral corticosteroids. Six of these seven patients were treated with biologic DMARDs because of refractoriness to conventional DMARDs. Three of the six treated with biologic DMARDs had a full improvement, whereas the other three had a partial improvement in cerebral ischemic symptoms following treatment [62]. In contrast to this, few patients in our cohort with TAK had been treated with biologic DMARDs. However, a majority were in clinical remission on follow-up. To date, no DMARD (conventional, biologic, or targeted synthetic) has been proven to be effective in a clinical trial of TAK patients. The choice of DMARDs in TAK patients is often empiric, based on observational data and clinical experience [3,23,60,63].

From those TAK patients in our cohort who had a stroke at presentation, 59% were on aspirin, 28% were on clopidogrel, and 45% were on statins. Ringleb et al. reported the use of aspirin in 60% of their cohort of TAK patients with stroke [61]. Couture et al. reported the use of antiplatelet agents in all, statins in 76%, and anticoagulation in 18% of their patients with TAK with stroke [32]. Goel et al. reported that among the sixteen patients with TAK with stroke at cohort entry, 50% were on antiplatelet drugs and 50% were on statins [8]. Mirouse et al. reported the use of aspirin in 40%, clopidogrel in 8%, anticoagulation in 10%, and statins in 16% of their TAK patients with stroke [34]. All patients with TAK with ischemic stroke might not be initiated on antiplatelet drugs or statins if the stroke was deemed inflammatory in origin related to active disease. This is supported by the fact that, in the cohort of Kong et al., 26% of TAK patients with cerebral ischemia developed as such despite being on antiplatelet drugs [33]. Anticoagulation in the context of stroke in TAK patients is warranted if associated with persistent circulating antiphospholipid antibodies, or if stroke occurs after cardiac valvular surgery, which necessitates anticoagulation. None of our TAK patients with stroke had received anticoagulation since none of them had tested positive for antiphospholipid antibodies or had undergone cardiac surgeries.

### 4.10. Survival in TAK Patients with Stroke/TIA

Stroke remains the second leading cause of death worldwide [64]. Delay in diagnosis adversely affects prognosis in different rheumatic diseases [41,42]. Differences in presentation and prognosis of TAK have been observed with gender, age at disease onset, and disease activity at presentation or on follow-up [19,43,44,45]. However, we observed similar mortality in our patients with TAK with or without stroke, even after adjustment or matching for the prognostic factors of gender, age at disease onset, delay to diagnosis, disease activity at baseline, and the use of conventional, biologic, or targeted synthetic DMARDs (as a surrogate of longitudinal disease activity). Couture et al. reported deaths in similar proportions of TAK patients with or without stroke (in 2/17 TAK with stroke and 1/17 without stroke) [32]. Yang et al. observed 3 deaths among their 30 patients with TAK with stroke [51]. de Paula et al. reported one death among their eighteen patients of TAK with stroke following cerebral revascularization surgery [47]. Hoffmann et al. reported one death among ten patients with TAK with stroke [46]. Therefore, based on the information derived from retrospective cohorts, stroke in TAK patients does not appear to increase the risk of death. As discussed earlier, stroke has been reported in TAK patients following vascular interventions [13,28,29,30]. However, none of our patients had developed a stroke after procedures related to TAK.

### 4.11. Limitations and Strengths of Our Study

The retrospective study design was a limitation that lends the possibility of recall bias in view of the reliance on the data available on the retrospective review of charts. However, the data was retrieved from clinic files at a dedicated vasculitis clinic in a tertiary care training and referral center. Since this was a single-center retrospective study, the study findings might have been affected by selection bias as well as treatment bias. Detailed findings on the imaging of intracranial vessels in patients with TAK with stroke/TIA were unavailable due to the retrospective data retrieval [65]. Patient-reported outcome measures (PROMs) in TAK patients with stroke compared to those without could not be compared given the retrospective cohort study design. Overall, few studies have reported PROMs in TAK patients [66], none of which had compared PROMs in TAK patients with or without stroke/TIA. While the sample size of 191 patients with TAK might be considered to be limited in number, it must be kept in mind that TAK is a rare form of LVV. Few multicentric studies have published findings on a similar number of patients with TAK [7,67]. The strengths of our study were the cohort of patients with TAK followed up in a dedicated vasculitis clinic with a mean follow-up duration exceeding 3.5 years. The number of observed strokes or TIAs in our patients with TAK, while overall a relatively small number, was large enough to permit meaningful comparisons with TAK patients without stroke or TIA. However, the disproportionate size of the groups of patients with TAK with or without stroke/TIA was a limitation. Unadjusted as well as adjusted or matched analyses for assessing mortality in our cohort of TAK patients with stroke or TIA lends confidence to our observation of similar mortality in TAK patients with or without stroke. However, the effect of unmeasured confounding variables on the outcome cannot be excluded due to the study design of a retrospective cohort [68]. The unavailability of data regarding the severity of stroke using validated indices such as the National Institutes of Health Stroke Scale meant that we could not adjust the risk of mortality with stroke in TAK patients with the severity of stroke [69].

### 4.12. Agenda for Future Research concerning Stroke/TIA in TAK Patients

Further prospective studies should attempt to validate the observation of similar mortality in TAK patients with or without stroke/TIA from the present retrospective study, while also adjusting for the severity of stroke using validated scales. Differences in patient-reported outcomes in TAK patients with or without stroke/TIA should be explored. Differences in genetic risk factors and other immune pathologies in TAK patients with or without stroke/TIA should also be elucidated.

## 5. Conclusions

Nearly one-sixth of the patients with TAK in this large retrospective cohort from North India had stroke or TIA at diagnosis or on follow-up. Ischemic strokes were more common than hemorrhagic strokes, and recurrent strokes were observed in 9% of patients. Those with stroke/TIA were more likely to be older or males, have vision loss, and more frequently had Hata’s angiographic type IIa than those without stroke/TIA. The overall use of immunosuppressive therapies was similar in TAK patients with or without stroke/TIA, other than cyclophosphamide, which was used more often used in those with stroke/TIA (albeit used sparingly overall). Stroke or TIA did not seem to adversely affect survival in unmatched or matched analyses. Future prospective studies of stroke/TIA in TAK patients should evaluate the quality of life and other PROMs in this subset of TAK patients.

## Figures and Tables

**Table 1:** Demographics of the cohort, prevalent comorbidities, baseline disease activity status, and mortality for TAK patients with or without stroke/TIA.

	TAK without Stroke/TIA (*n* = 157)	TAK with Stroke/TIA (*n* = 34)	*p* Value *
**Demographic characteristics**			
Age at cohort entry Mean (±SD)	24.74 ± 10.06	28.65 ± 10.74	**0.044**
Sex distribution (Female: Male)	122:35	20:14	**0.022 ^a^**
Diagnostic delay (years) Mean (±SD)	3.08 ± 3.93 (*n* = 155)	2.70 ± 4.15	0.606
Duration of follow-up (months) Mean (±SD)	43.26 ± 44.92	59.29 ± 70.32	0.094
**Prevalence of recorded comorbidities**			
Diabetes mellitus	2 (1.27%)	4 (11.76%)	**0.010 ^b^**
Smoking	1 (0.64%)	1 (2.94%)	0.325 ^b^
Dyslipidemia	0 (0%)	0 (0%)	-
Cancer	0 (0%)	0 (0%)	-
Other autoimmune diseases	3 (1.91%)	0 (0%)	>0.999 ^b^
	Statin-induced necrotising myositis (*n* = 1) Systemic sclerosis (*n* = 1) Crohn’s disease (*n* = 1)	-	-
Other comorbidities	11 (7.01%)	2 (5.88%)	>0.999 ^b^
	Hypothyroidism (*n* = 8) Epilepsy (*n* = 1) Osteoporosis (*n* = 1) Rheumatic heart disease (*n* = 1)	Hypothyroidism (*n* = 2)	-
**Disease activity and outcomes**			
DEI.TAK score at first visit (mean (±SD))	9.54 ± 6.42	8.45 ± 5.33 (*n* = 31) **	0.377
ITAS2010 at first visit (mean (±SD))	11.07 ± 7.43	10.55 ± 6.29 (*n* = 31) **	0.715
Proportion with active disease at first visit	127 (80.89%)	24 (77.42%) (*n* = 31) **	0.657 ^a^
Mortality (*n*, %)	8 (5.10%)	2 (5.88%)	0.693 ^b^

* Unpaired t-test for mean (SD), Chi squared ^a^ /Fisher’s exact ^b^ for proportions; ** TAK with stroke/TIA at presentation DEI.TAK—Disease Extent Index in Takayasu arteritis; ITAS2010—Indian Takayasu arteritis Clinical Activity Score; SD—Standard deviation; *p* values < 0.05 are highlighted in bold.

**Table 2:** Clinical features and blood investigations at presentation for TAK patients with or without stroke/TIA.

	TAK without Stroke/TIA (*n* = 157)	TAK with Stroke/TIA (*n* = 34)	Odds Ratio (95%CI) (with vs. without Stroke/TIA)	*p* Value *
**Clinical features at presentation (*n*(%))**				
Constitutional (Fever, fatigue, weight loss, myalgia, arthralgia/arthritis taken together)	71 (45.22%)	10 (29.41%)	0.51 (0.23–1.13)	0.091 ^a^
Carotidynia	20 (12.74%)	1 (2.94%)	0.21 (0.03–1.60)	0.132 ^b^
Headache	26 (16.56%)	3 (8.82%)	0.49 (0.14–1.71)	0.305 ^b^
Syncope/Dizziness/Vertigo (taken together)	31 (19.75%)	6 (17.65%)	0.87 (0.33–2.29)	0.779 ^a^
Seizure	6 (3.82%)	3 (8.82%)	2.44 (0.58–10.27)	0.202 ^b^
Blurring vision	20 (12.74%)	4 (11.76%)	0.91 (0.29–2.87)	>0.999 ^b^
Loss of vision	5 (3.21%)	5 (14.71%)	5.21 (1.42–19.14)	**0.007 ^a^**
Documented TAK retinopathy	2 (1.27%)	0 (0%)	-	>0.999 ^b^
Inflammatory ocular disease	2 (1.27%)	0 (0%)	-	>0.999 ^b^
Pulse or BP inequality	77 (49.04%)	10 (29.41%)	0.43 (0.19–0.96)	**0.037 ^a^**
Pulse loss	100 (63.69%)	22 (64.71%)	1.05 (0.48–2.27)	0.911 ^a^
Vascular bruits	115 (73.25%)	20 (58.82%)	0.52 (0.24–1.13)	0.094 ^a^
Claudication (upper or lower limb)	65 (41.40%)	10 (29.41%)	0.59 (0.26–1.32)	0.194 ^a^
Hypertension	128 (81.53%)	25 (73.53%)	0.63 (0.27–1.49)	0.289 ^a^
Aortic regurgitation	10 (6.37%)	1 (2.94%)	0.45 (0.06–3.60)	0.692 ^b^
Renal failure (acute or chronic)	14 (8.92%)	2 (5.88%)	0.64 (0.14–2.95)	0.742 ^b^
Abdominal angina	7 (4.46%)	0 (0%)	-	0.356 ^b^
Bowel infarcts	1 (0.64%)	0 (0%)	-	>0.999 ^b^
Chest pain	16 (10.19%)	1 (2.94%)	0.27 (0.03–2.09)	0.316 ^b^
Acute coronary syndrome	0 (0%)	0 (0%)	-	-
Heart failure	20 (12.74%)	2 (5.88%)	0.43 (0.10–1.93)	0.377 ^b^
Erythema nodosum	2 (1.27%)	0 (0%)	-	>0.999 ^b^
Other	21 (13.38%)	1 (2.94%)	-	-
	Breathlessness (*n* = 8) Palpitations (*n* = 8) Hemoptysis (*n* = 2) Digital gangrene (*n* = 1) Left upper limb numbness (*n* = 1) Oral ulcers (*n* = 1)	Breathlessness (*n* = 1)	-	-
**Baseline investigations**				
ESR (mm/h) (mean (±SD))	49.10 ± 32.34 (*n* = 153)	30.52 ± 23.85 (*n* = 33)	-	**0.002**
CRP (mg/L) (mean (±SD))	24.53 ± 32.72 (*n* = 147)	20.23 ± 28.57 (*n* = 32)	-	0.493
Hemoglobin (g/dL) (mean (±SD))	11.18 ± 2.03 (*n* = 153)	12.33 ± 1.76 (*n* = 32)	-	**0.003**
Neutrophil: Lymphocyte ratio	3.29 ± 1.72 (*n* = 141)	3.32 ± 4.02 (*n* = 32)	-	0.937

* Chi squared ^a^ /Fisher’s exact ^b^ for proportions; BP—Blood pressure; 95%CI—95% confidence interval; CRP—C-reactive protein; ESR—Erythrocyte sedimentation rate; SD—Standard deviation; TAK—Takayasu arteritis; TIA—Transient ischemic attack. *p* values < 0.05 are highlighted in bold.

**Table 3:** Imaging modalities and angiographic subtypes at the initial assessment for TAK patients with or without stroke/TIA.

	TAK without Stroke/TIA (*n* (%))	TAK with Stroke/TIA (*n* (%))	Odds Ratio (95%CI) (with vs. without Stroke/TIA)	*p* Value *
**Imaging modalities**	*n* = 157	*n* = 34		
PET-CT	46 (29.30%)	16 (47.06%)	2.14 (1.01–4.57)	**0.045 ^a^**
CT angiography	118 (75.16%)	25 (73.53%)	0.92 (0.39–2.13)	0.843 ^a^
MR angiography	38 (24.20%)	12 (35.29%)	1.71 (0.77–3.77)	0.182 ^a^
Conventional angiography	16 (10.19%)	6 (17.65%)	1.89 (0.68–5.25)	0.217 ^a^
Ultrasound	12 (7.64%)	3 (8.82%)	1.17 (0.31–4.39)	0.734 ^b^
**Angiographic subtype**	*n* = 157	*n* = 34		
Hata I	18 (11.46%)	7 (20.59%)	2.00 (0.76–5.26)	0.153 ^a^
Hata IIa	3 (1.91%)	6 (17.65%)	11.00 (2.60–46.58)	**0.001 ^b^**
Hata IIb	15 (9.55%)	5 (14.71%)	1.63 (0.55–4.85)	0.374 ^a^
Hata III	9 (5.73%)	1 (2.94%)	0.50 (0.06–4.07)	>0.999 ^b^
Hata IV	11 (7.01%)	4 (11.76%)	1.77 (0.53–5.93)	0.313 ^b^
Hata V	101 (64.33%)	11 (32.35%)	0.27 (0.12–0.58)	**0.001 ^a^**
Hata P+	10 (6.37%)	1 (2.94%)	0.45 (0.06–3.60)	0.692 ^b^
Hata C+	3 (1.91%)	0 (0%)	-	>0.999 ^b^

* Chi squared ^a^ /Fisher’s exact ^b^ for proportions; 95%CI—95% confidence interval; C+—Coronary involvement; CT—Computerized tomography; PET—Positron emission tomography; P+—Pulmonary involvement; *p* values < 0.05 are highlighted in bold.

**Table 4:** Comparison of the choice of first- and second-line disease-modifying anti-rheumatic drugs among TAK patients with or without stroke/TIA.

	TAK without Stroke/TIA (*n* = 157)	TAK with Stroke/TIA (*n* = 34)
First-line DMARD	*n* = 118 Methotrexate (*n* = 56) Tacrolimus (*n* = 42) Mycophenolate (*n* = 9) Azathioprine (*n* = 8) Cyclophosphamide (*n* = 1) Methotrexate+Mycophenolate (*n* = 1) Tocilizumab (*n* = 1)	*n* = 25 Methotrexate (*n* = 8) Tacrolimus (*n* = 8) Azathioprine (*n* = 4) Mycophenolate (*n* = 3) Cyclophosphamide followed by azathioprine (*n* = 2)
Second-line DMARD (switch or add-on)	*n* = 44 Mycophenolate (*n* = 14) Azathioprine (*n* = 10) Methotrexate (*n* = 8) Tacrolimus (*n* = 8) Tocilizumab (*n* = 2) Adalimumab (*n* = 1) Leflunomide (*n* = 1)	*n* = 6 Methotrexate (*n* = 2) Azathioprine (*n* = 1) Cyclophosphamide (*n* = 1) Leflunomide (*n* = 1) Mycophenolate (*n* = 1)

DMARD—Disease-modifying anti-rheumatic drug.

**Table 5:** Comparison of disease activity at 6, 12 and 24 months of follow-up between TAK patients with or without stroke/TIA *.

Time Point	Disease Activity Assessment Tool	TAK without Stroke/TIA	TAK with Stroke/TIA	*p* Value **
6 months (*n* = 92 without stroke/TIA, 19 with stroke/TIA)	DEI.TAK	0.89 ± 1.28	0.53 ± 0.96	0.243
	ITAS2010	1.14 ± 1.70	0.74 ± 1.37	0.333
	Number of NIH disease activity criteria fulfilled	0.72 ± 0.73	0.58 ± 0.51	0.434
	Active as per PGA	20 (21.74%)	1 (5.26%)	0.081
12 months (*n* = 78 without stroke/TIA, 13 with stroke/TIA)	DEI.TAK	0.62 ± 1.01	0.39 ± 0.77	0.434
	ITAS2010	0.83 ± 1.44	0.46 ± 0.97	0.372
	Number of NIH disease activity criteria fulfilled	0.82 ± 0.68	0.38 ± 0.51	**0.030**
	Active as per PGA	21 (26.92%)	1 (7.69%)	0.121
24 months (*n* = 65 without stroke/TIA, 9 with stroke/TIA)	DEI.TAK	0.62 ± 1.09	1.33 ± 2.35	0.121
	ITAS2010	0.83 ± 1.55	1.78 ± 3.35	0.151
	Number of NIH disease activity criteria fulfilled	0.77 ± 0.68	0.67 ± 0.71	0.674
	Active as per PGA	11 (16.92%)	2 (22.22%)	0.497

DEI.TAK—Disease Extent Index in Takayasu arteritis; ITAS2010—Indian Takayasu arteritis Clinical Activity Score; NIH—National Institutes of Health; PGA—Physician global assessment. * Only those TAK patients with stroke or TIA at baseline were taken for this analysis; ** Unpaired Student’s t-test for continuous variables, Fisher’s exact test for categorical variables.

**Table 6:** Risk estimates for the association of stroke/TIA in TAK patients with mortality.

Model	Covariates Adjusted for in Cox Regression Model	Hazard Ratio (95% Confidence Intervals)
Exposure—TAK with stroke/TIA vs. without stroke/TIA Outcome—Mortality	None	0.76 (0.15–3.99)
	Gender (male or female)	0.56 (0.09–3.28)
	Gender (male or female), age at disease onset	0.83 (0.12–5.90)
	Gender (male or female), age at disease onset, delay to diagnosis	0.91 (0.12–6.62)
	Gender (male or female), age at disease onset, delay to diagnosis, whether disease was active at baseline assessment or not	1.36 (0.18–10.33)
	Gender (male or female), age at disease onset, delay to diagnosis, whether disease was active at baseline assessment or not, number of conventional DMARDs used	1.14 (0.15–8.89)
	Gender (male or female), age at disease onset, delay to diagnosis, whether disease was active at baseline assessment or not, number of conventional DMARDs used, number of biologic DMARDs used	1.38 (0.19–10.20)

*n* = 182 for all models; DMARDs—Disease-modifying anti-rheumatic drugs.

## Data Availability

All the analyses performed for this article have been reported in the main text or in the supplementary files. Data pertaining to the article shall be shared on reasonable request to the corresponding author (Durga Prasanna Misra, durgapmisra@gmail.com).

## Supplementary Materials

The following supporting information can be downloaded at: https://www.mdpi.com/article/10.3390/life12111904/s1, Table S1: Individual vascular involvement at initial assessment compared between TAK with or without stroke/TIA; Table S2: Treatments received by the cohort compared between TAK with or without stroke/TIA; Table S3: Summary of observational studies comparing patients with TAK with or without stroke or TIA.

## Abbreviations

AUC	Area under the receiver operating characteristics curve
CRP	C-reactive protein
CTA	Computerized tomographic angiography
CVD	Cardiovascular Disease
DEI.TAK	Disease Extent Index in TAK
DMARDs	Disease-modifying antirheumatic drugs
ESR	Erythrocyte sedimentation rate
GCA	Giant Cell Arteritis
IEC	Institute Ethics Committee
ITAS2010	Indian TAK Clinical Activity Score 2010
LVV	Large vessel vasculitis
MRI	Magnetic resonance imaging
MRA	Magnetic resonance angiography
NA	Not assessable
NIH	National Institutes of Health
PGA	Physician global assessment
PROMs	Patient-reported outcome measures
PET-CT	Positron emission tomography-computerized tomography
SD	Standard deviation
TAK	Takayasu arteritis
TIA	Transient ischemic attack

## References

[1] Pugh D., Karabayas M., Basu N., Cid M.C., Goel R., Goodyear C.S., Grayson P.C., McAdoo S.P., Mason J.C., Owen C. (2022). Large-vessel vasculitis. Nat. Rev. Dis. Primers.

[2] Watts R.A., Hatemi G., Burns J.C., Mohammad A.J. (2022). Global epidemiology of vasculitis. Nat. Rev. Rheumatol..

[3] Misra D.P., Wakhlu A., Agarwal V., Danda D. (2019). Recent advances in the management of Takayasu arteritis. Int. J. Rheum. Dis..

[4] Kermani T.A. (2019). Takayasu arteritis and giant cell arteritis: Are they a spectrum of the same disease?. Int. J. Rheum. Dis..

[5] Sakaue S., Hagino N. (2016). IMAGES IN CLINICAL MEDICINE. Takayasu’s Arteritis. N. Engl. J. Med..

[6] Singh K., Rathore U., Rai M.K., Behera M.R., Jain N., Ora M., Bhadauria D., Sharma S., Pande G., Gambhir S. (2022). Novel Th17 Lymphocyte Populations, Th17.1 and PD1+Th17, are Increased in Takayasu Arteritis, and Both Th17 and Th17.1 Sub-Populations Associate with Active Disease. J. Inflamm. Res..

[7] Quinn K.A., Gribbons K.B., Carette S., Cuthbertson D., Khalidi N.A., Koening C.L., Langford C.A., McAlear C.A., Monach P.A., Moreland L.W. (2020). Patterns of clinical presentation in Takayasu’s arteritis. Semin. Arthritis Rheum..

[8] Goel R., Chandan J.S., Thayakaran R., Adderley N.J., Nirantharakumar K., Harper L. (2021). Cardiovascular and Renal Morbidity in Takayasu Arteritis: A Population-Based Retrospective Cohort Study From the United Kingdom. Arthritis Rheumatol..

[9] Ahn S.S., Han M., Park Y.B., Jung I., Lee S.W. (2022). Incidence, prevalence and risk of stroke in patients with Takayasu arteritis: A nationwide population-based study in South Korea. Stroke Vasc. Neurol..

[10] Kang Z., Xu Z., Wu X., Nie C., Yin J., Mei B. (2021). Ischemic stroke as the first manifestation of Takayasu arteritis: High resolution magnetic resonance imaging. Neurol. Sci..

[11] Chiew Y.R., Seet Y.H.C. (2022). An Unusual Case of Stroke as the Initial Manifestation of Early Takayasu Arteritis with Normal Erythrocyte Sedimentation Rate (ESR): Diagnosis and Treatment. Am. J. Case Rep..

[12] Sasannejad P., Verdipour M., Asadi M., Ahmadi H. (2019). Recurrent ischemic stroke in a case of Takayasu’s arteritis, mimicking multiple sclerosis. Iran. J. Neurol..

[13] Fields C.E., Bower T.C., Cooper L.T., Hoskin T., Noel A.A., Panneton J.M., Sullivan T.M., Gloviczki P., Cherry K.J. (2006). Takayasu’s arteritis: Operative results and influence of disease activity. J. Vasc. Surg..

[14] Qi Y., Yang L., Zhang H., Liang E., Song L., Cai J., Jiang X., Zou Y., Qian H., Wu H. (2018). The presentation and management of hypertension in a large cohort of Takayasu arteritis. Clin. Rheumatol..

[15] Wong S.P.Y., Mok C.C., Lau C.S., Yip M.L., Tam L.S., Ying K.Y., Ng W.L., Ng K.H., Leung M.H., Lee T.Y. (2018). Clinical presentation, treatment and outcome of Takayasu’s arteritis in southern Chinese: A multicenter retrospective study. Rheumatol. Int..

[16] Newton L., Justice E., Carruthers D. (2010). Takayasu’s Arteritis: An important cause of stroke in younger patients. Acute Med..

[17] Smajlović D. (2015). Strokes in young adults: Epidemiology and prevention. Vasc. Health Risk. Manag..

[18] Karabacak M., Kaymaz-Tahra S., Şahin S., Yıldız M., Adroviç A., Barut K., Direskeneli H., Kasapçopur Ö., Alibaz-Öner F. (2021). Childhood-onset versus adult-onset Takayasu arteritis: A study of 141 patients from Turkey. Semin. Arthritis Rheum..

[19] Misra D.P., Rathore U., Kopp C.R., Patro P., Agarwal V., Sharma A. (2022). Presentation and clinical course of pediatric-onset versus adult-onset Takayasu arteritis-a systematic review and meta-analysis. Clin. Rheumatol..

[20] Kerr G.S., Hallahan C.W., Giordano J., Leavitt R.Y., Fauci A.S., Rottem M., Hoffman G.S. (1994). Takayasu arteritis. Ann. Intern. Med..

[21] Mustafa K.N., Hadidy A., Sweiss N.J. (2010). Clinical and radiological features of Takayasu’s arteritis patients in Jordan. Rheumatol. Int..

[22] Jang S.Y., Park T.K., Kim D.K. (2021). Survival and causes of death for Takayasu’s arteritis in Korea: A retrospective population-based study. Int. J. Rheum. Dis..

[23] Rathore U., Thakare D.R., Patro P., Agarwal V., Sharma A., Misra D.P. (2022). A systematic review of clinical and preclinical evidences for Janus kinase inhibitors in large vessel vasculitis. Clin. Rheumatol..

[24] Misra D.P., Pande G., Agarwal V. (2022). Cardiovascular risks associated with Janus kinase inhibitors: Peering outside the black box. Clin. Rheumatol..

[25] Johnson A., Emery D., Clifford A. (2021). Intracranial Involvement in Takayasu’s Arteritis. Diagnostics.

[26] Hwang J., Kim S.J., Bang O.Y., Chung C.S., Lee K.H., Kim D.K., Kim G.M. (2012). Ischemic stroke in Takayasu’s arteritis: Lesion patterns and possible mechanisms. J. Clin. Neurol..

[27] Ham S.W., Kumar S.R., Rowe V.L., Weaver F.A. (2011). Disease progression after initial surgical intervention for Takayasu arteritis. J. Vasc. Surg..

[28] Diao Y.P., Chen Y.X., Yan S., Chen Z.G., Miao Q., Liu X.R., Liu C.W., Li Y.J. (2016). Efficacy and safety analysis of surgical bypass and endovascular management in the treatment of 116 Takayasu arteritis. Zhonghua Yi Xue Za Zhi.

[29] Saadoun D., Lambert M., Mirault T., Resche-Rigon M., Koskas F., Cluzel P., Mignot C., Schoindre Y., Chiche L., Hatron P.Y. (2012). Retrospective analysis of surgery versus endovascular intervention in Takayasu arteritis: A multicenter experience. Circulation.

[30] Jung J.H., Lee Y.H., Song G.G., Jeong H.S., Kim J.H., Choi S.J. (2018). Endovascular Versus Open Surgical Intervention in Patients with Takayasu’s Arteritis: A Meta-analysis. Eur. J. Vasc. Endovasc. Surg..

[31] Anusheel, Canenguez Benitez J.S., Jaka S., Roshan N.S., Kommuru S., Ahmed S., Kaur G., Desai N. (2022). Relationship Between Cerebrovascular Diseases and Vasculitis: A Cross-Sectional Nationwide Inpatient Study. Cureus.

[32] Couture P., Chazal T., Rosso C., Haroche J., Léger A., Hervier B., Deltour S., Amoura Z., Cohen Aubart F. (2018). Cerebrovascular events in Takayasu arteritis: A multicenter case-controlled study. J. Neurol..

[33] Kong F., Huang X., Su L., Liao Q., Wang C., Zhao Y. (2021). Risk factors for cerebral infarction in Takayasu arteritis: A single-centre case-control study. Rheumatology.

[34] Mirouse A., Deltour S., Leclercq D., Squara P.A., Pouchelon C., Comarmond C., Kahn J.E., Benhamou Y., Mirault T., Mekinian A. (2022). Cerebrovascular Ischemic Events in Patients With Takayasu Arteritis. Stroke.

[35] Arend W.P., Michel B.A., Bloch D.A., Hunder G.G., Calabrese L.H., Edworthy S.M., Fauci A.S., Leavitt R.Y., Lie J.T., Lightfoot R.W. (1990). The American College of Rheumatology 1990 criteria for the classification of Takayasu arteritis. Arthritis Rheum..

[36] Jennette J.C., Falk R.J., Bacon P.A., Basu N., Cid M.C., Ferrario F., Flores-Suarez L.F., Gross W.L., Guillevin L., Hagen E.C. (2013). 2012 revised International Chapel Hill Consensus Conference Nomenclature of Vasculitides. Arthritis Rheum..

[37] Ozen S., Pistorio A., Iusan S.M., Bakkaloglu A., Herlin T., Brik R., Buoncompagni A., Lazar C., Bilge I., Uziel Y. (2010). EULAR/PRINTO/PRES criteria for Henoch-Schonlein purpura, childhood polyarteritis nodosa, childhood Wegener granulomatosis and childhood Takayasu arteritis: Ankara 2008. Part II: Final classification criteria. Ann. Rheum. Dis..

[38] Hata A., Noda M., Moriwaki R., Numano F. (1996). Angiographic findings of Takayasu arteritis: New classification. Int. J. Cardiol..

[39] Aydin S.Z., Yilmaz N., Akar S., Aksu K., Kamali S., Yucel E., Karadag O., Bicakcigil M., Ozer H., Kiraz S. (2010). Assessment of disease activity and progression in Takayasu’s arteritis with Disease Extent Index-Takayasu. Rheumatology.

[40] Misra R., Danda D., Rajappa S.M., Ghosh A., Gupta R., Mahendranath K.M., Jeyaseelan L., Lawrence A., Bacon P.A. (2013). Development and initial validation of the Indian Takayasu Clinical Activity Score (ITAS2010). Rheumatology.

[41] Kernder A., Richter J.G., Fischer-Betz R., Winkler-Rohlfing B., Brinks R., Aringer M., Schneider M., Chehab G. (2021). Delayed diagnosis adversely affects outcome in systemic lupus erythematosus: Cross sectional analysis of the LuLa cohort. Lupus.

[42] Seo M.R., Baek H.L., Yoon H.H., Ryu H.J., Choi H.J., Baek H.J., Ko K.P. (2015). Delayed diagnosis is linked to worse outcomes and unfavourable treatment responses in patients with axial spondyloarthritis. Clin. Rheumatol..

[43] Mirouse A., Biard L., Comarmond C., Lambert M., Mekinian A., Ferfar Y., Kahn J.E., Benhamou Y., Chiche L., Koskas F. (2019). Overall survival and mortality risk factors in Takayasu’s arteritis: A multicenter study of 318 patients. J. Autoimmun..

[44] Yang L., Zhang H., Jiang X., Zou Y., Qin F., Song L., Guan T., Wu H., Xu L., Liu Y. (2014). Clinical manifestations and longterm outcome for patients with Takayasu arteritis in China. J. Rheumatol..

[45] Misra D.P., Aggarwal A., Lawrence A., Agarwal V., Misra R. (2015). Pediatric-onset Takayasu’s arteritis: Clinical features and short-term outcome. Rheumatol. Int..

[46] Hoffmann M., Corr P., Robbs J. (2000). Cerebrovascular findings in Takayasu disease. J. Neuroimaging.

[47] de Paula L.E., Alverne A.R., Shinjo S.K. (2013). Clinical and vascular features of Takayasu arteritis at the time of ischemic stroke. Acta Reumatol. Port..

[48] Arnaud L., Haroche J., Limal N., Toledano D., Gambotti L., Chalumeau N.C., Boutin D., Cacoub P., Cluzel P., Koskas F. (2010). Takayasu arteritis in France: A single-center retrospective study of 82 cases comparing white, North African, and black patients. Medicine.

[49] Li-xin Z., Jun N., Shan G., Bin P., Li-ying C. (2011). Neurological manifestations of Takayasu arteritis. Chin. Med. Sci. J..

[50] Soto M.E., Espinola N., Flores-Suarez L.F., Reyes P.A. (2008). Takayasu arteritis: Clinical features in 110 Mexican Mestizo patients and cardiovascular impact on survival and prognosis. Clin. Exp. Rheumatol..

[51] Yang L., Zhang H., Jiang X., Song L., Qin F., Zou Y., Wu H., Bian J., Zhou X., Hui R. (2015). Clinical Features and Outcomes of Takayasu Arteritis with Neurological Symptoms in China: A Retrospective Study. J. Rheumatol..

[52] Fan L., Zhang H., Cai J., Yang L., Wei D., Yu J., Fan J., Song L., Ma W., Lou Y. (2019). Clinical Course, Management, and Outcomes of Pediatric Takayasu Arteritis Initially Presenting With Hypertension: A 16-year overview. Am. J. Hypertens.

[53] Duarte M.M., Geraldes R., Sousa R., Alarcão J., Costa J. (2016). Stroke and Transient Ischemic Attack in Takayasu’s Arteritis: A Systematic Review and Meta-analysis. J. Stroke Cerebrovasc. Dis..

[54] Kim H., Barra L. (2018). Ischemic complications in Takayasu’s arteritis: A meta-analysis. Semin. Arthritis Rheum..

[55] Hoffman G.S. (1996). Takayasu arteritis: Lessons from the American National Institutes of Health experience. Int. J. Cardiol..

[56] Misra D.P., Jain N., Ora M., Singh K., Agarwal V., Sharma A. (2022). Outcome Measures and Biomarkers for Disease Assessment in Takayasu Arteritis. Diagnostics.

[57] Schreiber K., Sciascia S., de Groot P.G., Devreese K., Jacobsen S., Ruiz-Irastorza G., Salmon J.E., Shoenfeld Y., Shovman O., Hunt B.J. (2018). Antiphospholipid syndrome. Nat. Rev. Dis. Primers..

[58] Jordan N.P., Bezanahary H., D’Cruz D.P. (2015). Increased risk of vascular complications in Takayasu’s arteritis patients with positive lupus anticoagulant. Scand. J. Rheumatol..

[59] Misra D.P., Rathore U., Patro P., Agarwal V., Sharma A. (2021). Corticosteroid monotherapy for the management of Takayasu arteritis-a systematic review and meta-analysis. Rheumatol. Int..

[60] Misra D.P., Rathore U., Patro P., Agarwal V., Sharma A. (2021). Disease-modifying anti-rheumatic drugs for the management of Takayasu arteritis—A systematic review and meta-analysis. Clin. Rheumatol..

[61] Ringleb P.A., Strittmatter E.I., Loewer M., Hartmann M., Fiebach J.B., Lichy C., Weber R., Jacobi C., Amendt K., Schwaninger M. (2005). Cerebrovascular manifestations of Takayasu arteritis in Europe. Rheumatology.

[62] Porter A., Youngstein T., Tombetti E., Mason J.C. (2020). Biologic therapy in supra-aortic Takayasu arteritis can improve symptoms of cerebral ischaemia without surgical intervention. Rheumatology.

[63] Misra D.P., Sharma A., Kadhiravan T., Negi V.S. (2017). A scoping review of the use of non-biologic disease modifying anti-rheumatic drugs in the management of large vessel vasculitis. Autoimmun. Rev..

[64] Collaborators G.S. (2021). Global, regional, and national burden of stroke and its risk factors, 1990-2019: A systematic analysis for the Global Burden of Disease Study 2019. Lancet Neurol..

[65] Guo Y., Du J., Li T., Gao N., Pan L. (2022). Clinical features and risk factors of intracranial artery disease in patients with Takayasu arteritis. Clin. Rheumatol..

[66] Misra D.P., Rathore U., Patro P., Agarwal V., Sharma A. (2021). Patient-Reported Outcome Measures in Takayasu Arteritis: A Systematic Review and Meta-Analysis. Rheumatol. Ther..

[67] Sağlam B., Kaymaz-Tahra S., Kenar G., Kocaer S., Omma A., Erden A., Kara M., Yazıcı A., Cefle A., Gerçik Ö. (2022). Metabolic syndrome is associated with increased cardiovascular risk and disease damage in patients with Takayasu arteritis. Int. J. Rheum. Dis..

[68] Vanderweele T.J., Arah O.A. (2011). Bias formulas for sensitivity analysis of unmeasured confounding for general outcomes, treatments, and confounders. Epidemiology.

[69] Fischer U., Baumgartner A., Arnold M., Nedeltchev K., Gralla J., De Marchis G.M., Kappeler L., Mono M.L., Brekenfeld C., Schroth G. (2010). What is a minor stroke?. Stroke.

