# Supplementary material for: Comparison of Presentation and Prognosis of Takayasu Arteritis with or without Stroke or Transient Ischemic Attack—A Retrospective Cohort Study
Source: Life (Basel). 2022 Nov 16;12(11):1904. doi: 10.3390/life12111904 (PMC9697956; doi:10.3390/life12111904)
Supplement: Supplementary file 1 [file life-12-01904-s001.zip › life-2032194-supplementary.pdf]

Supplementary tables

Table S1. Individual vascular involvement at initial assessment compared between TAK with or without stroke/TIA

	**TAK without Stroke/TIA [*n* (%)]**	**TAK with Stroke/TIA [*n* (%)]**	**Odds Ratio (95% CI) (with vs. without stroke/TIA)**	***p* Value ***
**Vascular involvement**	*n* = 157	*n* = 33		
Coronary	3 (1.91%)	0 (0%)	-	>0.999 [b]
Right subclavian	56 (35.67%)	19 (57.58%)	2.45 (1.14–5.25)	**0.019** [a]
Left subclavian	114 (72.61%)	24 (72.73%)	1.01 (0.43–2.34)	0.989 [a]
Right carotid	56 (35.67%)	19 (57.58%)	2.45 (1.14–5.25)	**0.019** [a]
Left carotid	79 (50.32%)	20 (60.61%)	1.52 (0.71–3.26)	0.282 [a]
Right vertebral	6 (3.82%)	0 (0%)	-	0.592 [b]
Left vertebral	18 (11.46%)	4 (12.12%)	1.07 (0.34–3.38)	>0.999 [b]
Pulmonary	10 (6.37%)	1 (3.03%)	0.46 (0.06–3.72)	0.693 [b]
Brachiocephalic	37 (23.57%)	11 (33.33%)	1.62 (0.72–3.65)	0.241 [a]
Ascending aorta	31 (19.75%)	5 (15.15%)	0.73 (0.26–2.03)	0.540 [a]
Arch of aorta	55 (35.03%)	12 (36.36%)	1.06 (0.49–2.31)	0.884 [a]
Descending thoracic aorta	85 (54.14%)	9 (27.27%)	0.32 (0.14–0.73)	**0.005** [a]
Abdominal aorta	95 (60.51%)	10 (30.30%)	0.28 (0.13–0.64)	**0.002** [a]
Celiac trunk	46 (29.30%)	3 (9.09%)	0.24 (0.07–0.83)	**0.016** [b]
Superior mesenteric artery	39 (24.84%)	5 (15.15%)	0.54 (0.20–1.50)	0.230 [a]
Inferior mesenteric artery	8 (5.10%)	0 (0%)	-	0.355 [b]

Right renal	69 (43.95%)	12 (36.36%)	0.73 (0.34–1.58)	0.423 [a]
Left renal	67 (42.68%)	11 (33.33%)	0.67 (0.30–1.48)	0.321 [a]
Right iliac	9 (5.73%)	3 (9.09%)	1.64 (0.42–6.43)	0.441 [b]
Left iliac	10 (6.37%)	3 (9.09%)	1.47 (0.38–5.66)	0.702 [b]
Right femoral	1 (0.64%)	1 (3.03%)	4.88 (0.30–79.99)	0.318 [b]
Left femoral	2 (1.27%)	1 (3.03%)	2.42 (0.21–27.52)	0.438 [b]

* Chi squared [a]/ Fisher's exact [b] for proportions
TAK—Takayasu arteritis
95% CI—95% confidence *p* values < 0.05 are highlighted in bold

Table S2. Treatments received by the cohort compared between TAK with or without stroke/TIA

	TAK without Stroke/TIA (n = 157)	TAK with Stroke/TIA (n = 34)	p Value *
**Glucocorticoids**			
n(%)	120 (76.92%)	28 (82.35%)	0.489 [a]
on intravenous methylprednisolone n(%)	2 (1.65%)	0 (0%)	>0.999 [b]
Starting dose (mean with SD)	33.73 ± 14.40 (n = 117)	30.67 ± 15.74 (n = 26)	0.337
Continuing at last follow-up n(%)	95 (79.17%)	23 (67.65%)	0.161 [a]
Duration in months (mean ± SD)	36.63 ± 31.76 (n = 117)	42.48 ± 55.14 (n = 23)	0.484
Percentage reduction in prednisolone at last visit	86.93 ± 17.65 (n = 101)	90.44 ± 13.16 (n = 18)	0.423
**Methotrexate**			
n(%)	69 (43.95%)	11 (32.35%)	0.214 [a]
Continuing at last follow-up n(%)	34 (49.28%)	6 (54.55%)	0.745 [a]
Duration in months (mean ± SD)	33.04 ± 33.94 (n = 67)	31.40 ± 21.26 (n = 10)	0.883
**Leflunomide**			
n (%)	1 (0.64%)	1 (2.94%)	0.325 [b]
Continuing at last follow-up n (%)	1 (100%)	0 (0%)	>0.999 [b]
Duration in months (mean ± SD)	3	13	-
**Azathioprine**			
n (%)	20 (12.74%)	8 (23.53%)	0.107 [a]
Continuing at last follow-up n (%)	6 (30.00%)	3 (37.50%)	>0.999 [b]
Duration in months (mean ± SD)	29.63 ± 29.22 (n = 19)	34.38 ± 46.98 (n = 7)	0.758
**Mycophenolate**			
n (%)	28 (17.83%)	6 (17.65%)	0.979 [a]
Continuing at last follow-up n(%)	16 (57.14%)	3 (50%)	>0.999 [b]

Duration in months (mean ± SD)	17.73 ± 17.65 (*n* = 28)	12.50 ± 12.32 (*n* = 6)	0.497
**Tacrolimus**			
n(%)	59 (37.58%)	9 (26.47%)	0.220 [a]
Continuing at last follow-up n(%)	46 (77.97%)	8 (88.89%)	0.450 [a]
Duration in months (mean ± SD)	16.66 ± 18.28 (*n* = 59)	27.67 ± 30.81 (*n* = 9)	0.133
**Cyclophosphamide**			
n(%)	1 (0.64%)	3 (8.82%)	**0.018** [b]
Continuing at last follow-up n(%)	0 (0%)	0 (0%)	-
Duration in months (mean ± SD)	6	5.67 ± 2.52	-
**Total number of csDMARDs received** (mean with SD)	1.13 ± 0.90	1.12 ± 1.12	0.928
**Adalimumab**			
n(%)	1 (0.64%)	0 (0%)	>0.999 [b]
Continuing at last follow-up n(%)	1 (100%)	-	-
Duration in months (mean ± SD)	10	-	-
**Tocilizumab**			
n(%)	3 (1.91%)	1 (2.94%)	0.547 [b]
Continuing at last follow-up n(%)	0 (0%)	0 (0%)	-
Duration in months (mean ± SD)	4.67 ± 0.58	26	-
**Tofacitinib**			
n(%)	0 (0%)	1 (2.94%)	0.178 [b]
Continuing at last follow-up n(%)	-	1 (100%)	-
Duration in months (mean ± SD)	-	12	-
**Total number of ts or bDMARDs received** (mean with SD)	0.03 ± 0.18	0.06 ± 0.34	0.508
**Antihypertensives**			
**n(%)**	124 (78.98%)	24 (70.59%)	0.288 [a]
Mean (± SD) number of antihypertensives at presentation	2.24 ± 1.17 (*n* = 123)	2.25 ± 1.22 (*n* = 24)	0.957

**Aspirin**	33 (21.02%)	15 (57.7%) [a]	**<0.001** [a]
**Clopidogrel** [a]	13 (8.28%)	7 (26.9%) [a]	**0.010** [a]
**Statin** [a]	10 (6.37%)	13 (50%) [a]	**<0.001** [a]

* Chi squared [a] /Fisher's exact [b] for proportions

[a] Amongst those with ischemic stroke at presentation (n = 26)

SD—Standard deviation; TAK—Takayasu arteritis

p values < 0.05 are highlighted in bold

Table S3. Summary of observational studies comparing patients with TAK with or without stroke or TIA.

Study, Country [Reference]	Number of Stroke/TIA, Number of Controls	Summary of Key Differences
Couture et al. 2018, France [32]	17 stroke/TIA, 17 sex-matched controls	TAK with vs. without stroke/TIA.<br>• Similar traditional CVD risk factors<br>• Similar clinical features related to TAK<br>• Similar extracranial vascular involvement<br>• Greater frequency of intracranial involvement<br>• Two deaths vs one death
Ahn et al. 2022, South Korea [9]	73 stroke, 992 without stroke	• SIR for stroke with TAK 7.39 (95%CI 5.79–9.29) when compared with the general population.<br>TAK with vs without stroke<br>• Similar age and gender distribution<br>• Similar traditional CVD risk factors
Kong et al. 2021, China [33]	42 TAK with cerebral infarction, 80 TAK without cerebral infarction	TAK with vs. without cerebral infarction<br>• Relatively greater proportion of males<br>• More frequent hyperlipidemia<br>• More common blurring of vision & syncope<br>• Higher baseline disease activity by ITAS2010.<br>• Similar distribution of Hata's angiographic subtypes<br>• More frequent involvement of common carotid artery, internal carotid artery, vertebral artery, basilar artery, anterior cerebral artery, middle cerebral artery, and posterior cerebral artery<br>• Greater number of stenosed arteries<br>• Greater number of occluded arteries.<br>ITAS2010 ≥ 6 had AUC of 0.692 for stroke<br>ITAS2010 ≥ 7 had AUC of 0.754 for stroke
Mirouse et al. 2022, France [34]	63 TAK with stroke or TIA, 257 without stroke or TIA	TAK with vs. without stroke/TIA<br>• Similar distribution of age and sex<br>• Longer delay to diagnosis<br>• Greater prevalence of carotidodynia<br>• More frequently had a prior history of stroke<br>• Less frequently had a prior history of myocardial infarction<br>• Greater frequency of involvement of supra-aortic trunk and thoracic aorta

Misra et al. 2022, India [present study]	34 stroke/TIA, 157 controls (34 matched pairs using propensity scores)	TAK with vs. without stroke/TIA<br>• Relatively greater proportion of males<br>• Older age<br>• More frequently had diabetes<br>• More often had vision loss or syncope<br>• Less often had asymmetry of pulse or blood pressure<br>• More often had Hata's angiographic subtype IIa and less frequently had subtype V.<br>• Similar mortality—unadjusted hazard ratio 0.76 (95%CI 0.15–3.99), hazard ratio adjusted for gender, age at onset, delay to diagnosis, disease activity at baseline, DMARD use 1.38 (95%CI 0.19–10.20).<br>• Similar mortality between propensity-score matched pairs of TAK with or without stroke/TIA.

95%CI—95% confidence interval; AUC—Area under the receiver operating characteristics curve; CVD—Cardiovascular disease; DMARD—Disease-modifying anti-rheumatic drug; ITAS2010—Indian Takayasu Arteritis Clinical Activity Score 2010; TAK—Takayasu arteritis; TIA—Transient Ischemic Attack
